# Root resorption in fixed versus clear aligner therapy: A CBCT-based comparative study

**DOI:** 10.6026/973206300214307

**Published:** 2025-12-15

**Authors:** Zeeshan Mubashir, Ravi Ranjan Sinha, Monika Koul, Panga Gnana Sarita Kumari, Rushit Patel, Afrah Fatima, Rahul Tiwari

**Affiliations:** 1 Department of Orthodontics and Dentofacial Orthopaedics, Al Jouf Specialised Dental Center, Ministry of Health, Sakaka, Aljouf Province, Kingdom of Saudi Arabia; 2 Department of Prosthodontics, The Oxford Dental College, Bengaluru, Karnataka, India; 3 Department of Orthodontics and Dentofacial Orthopaedics, M.M. College of Dental Sciences and Research, Maharishi Markandeshwar (Deemed-to-be University), Mullana, Ambala, Haryana, India; 4 Department of Public Health Dentistry, SIBAR Institute of Dental Sciences, Takkellapadu, Guntur andhra Pradesh, India; 5 Department of Oral & Maxillofacial Surgery, Karnavati School of Dentistry, Uvarsad, Gandhinagar, Gujarat, India; 6 Department of Orthodontics, Panineeya Institute of Dental Sciences and Research Centre, Hyderabad, Telangana, India; 7 Department of Oral and Maxillofacial Surgery, RKDF Dental College and Research Centre, Sarvepalli Radhakrishnan University, Bhopal, Madhya Pradesh, India

**Keywords:** Root resorption, orthodontic appliances, fixed, orthodontic appliances, removable, cone-beam computed tomography, tooth movement

## Abstract

Root resorption is an undesirable but common sequela of orthodontic tooth movement. Therefore, it is of interest to compare root resorption associated with fixed orthodontic appliances and clear aligner therapy using cone-beam computed tomography (CBCT). Data revealed significantly higher external apical root resorption in fixed appliance patients compared to aligner users. Thus, CBCT provided precise volumetric measurements, confirming aligners as a safer modality for minimizing root resorption.

## Background:

Root resorption is an undesirable but common sequela of orthodontic tooth movement. It is characterized by the loss of cementum and dentin at the root apex due to the pressure and cellular response associated with mechanical forces. The clinical concern arises from its potential to compromise tooth stability and long-term prognosis [1]. Orthodontic appliances differ in biomechanics, force magnitude and duration of force application, which influence the extent of root resorption. Fixed orthodontic therapy has long been associated with higher rates of external apical root resorption (EARR) due to continuous and often heavier forces applied to teeth [2]. In contrast, clear aligner therapy applies intermittent and lighter forces, which may result in less root resorption [3].

Cone-beam computed tomography (CBCT) has emerged as a precise tool to evaluate root resorption three-dimensionally. Unlike periapical or panoramic radiographs, CBCT provides volumetric measurements, allowing accurate quantification of apical changes [4, 5-6]. Previous studies have reported variable findings. Some found no difference between appliance types, while others confirmed greater resorption in fixed therapies. The lack of standardized imaging and sample heterogeneity limits conclusions [7]. Therefore, it is of interest to report to compare root resorption between fixed orthodontics and clear aligner therapies using CBCT in a standardized cohort.

## Materials and Methods:

## Study design:

This retrospective comparative study was conducted at a tertiary orthodontic center. Ethical approval was obtained from the institutional review board and all subjects had provided informed consent for the use of their records.

## Sample selection:

A total of 60 patients (n=30 fixed appliances, n=30 clear aligners) aged 18-30 years were included. Inclusion criteria:

[1] Mild to moderate crowding (2-6 mm).

[2] No history of previous orthodontic treatment.

[3] Complete CBCT records before and after treatment.

## Exclusion criteria:

[1] Systemic diseases affecting bone metabolism.

[2] History of trauma or root anomalies.

[3] Incomplete root formation at baseline.

## Imaging protocol:

CBCT scans were acquired using the same machine (voxel size 0.25 mm, 90 kV, 10 mA). Pre- and post-treatment scans were evaluated. Images were analyze with *in vivo* 5.4 (Anatomage, USA) software.

## Measurements:

Root resorption was assessed for maxillary central and lateral incisors, premolars and first molars. Linear measurements (root length) and volumetric loss (mm^3^) was calculated. Apical root length was measured from the cement enamel junction (CEJ) to the apex.

## Statistical analysis:

Data were analyzed using SPSS v25.0.

[1] Paired t-test: intragroup pre- vs. post-treatment.

[2] Independent t-test: intergroup comparison.

[3] Significance level set at p<0.05.

## Results:

Both groups were comparable in age, gender distribution and treatment duration (fixed: 20.5 ± 3.2 months, aligners: 21.2 ± 2.9 months; p>0.05). Table 1 (see PDF) demonstrates a clear difference in root length reduction between the two groups. Patients treated with fixed appliances showed significantly greater apical shortening compared to those treated with clear aligners. Central and lateral incisors were most affected, with mean reductions exceeding 1 mm in the fixed group versus less than 0.7 mm in the aligner group. Premolars and molars also showed noticeable differences, though to a lesser degree. These findings confirm aligners limit linear apical shortening effectively. Table 2 (see PDF) illustrates volumetric root resorption across different tooth groups. Fixed appliance therapy resulted in markedly higher root volume loss, particularly in incisors, where mean loss exceeded 6 mm^3^. In contrast, aligner-treated patients demonstrated resorption values closer to 2 mm^3^ for incisors, suggesting substantially less apical damage. Premolars and molars showed similar patterns, though with lower absolute values. Overall, volumetric analysis reinforced that fixed appliances exert greater biological impact on root integrity than aligners.

## Discussion:

The present study compared root resorption between fixed orthodontics and clear aligner therapy using CBCT. The results demonstrated significantly greater resorption in fixed appliances, consistent with prior literature [8- 9]. Fixed appliances deliver continuous forces, often exceeding the optimal range for bone remodeling. This can cause localized ischemia, cementoblast damage and clastic activity, leading to root resorption [10]. In contrast, aligners exert intermittent, low-magnitude forces, allowing periods of tissue recovery and minimizing root damage [11]. Traditional 2D radiographs underestimate root resorption due to projection errors and superimposition. CBCT offers high-resolution, three-dimensional visualization, enabling accurate linear and volumetric assessment. Previous studies emphasized the superiority of CBCT in evaluating orthodontic-induced resorption [12]. Findings align with research showing central and lateral incisors as most prone to resorption. This is attributed to their long, tapered roots and susceptibility to torque movements. Similar comparative CBCT studies confirmed greater resorption with fixed appliances, reinforcing the current results [13]. However, some investigations reported no significant difference, possibly due to variation in treatment protocols, aligner wear compliance and imaging methodologies [14]. Differences in voxel size and volumetric calculation techniques can influence findings. The evidence suggests clear aligners are preferable for patients at high risk of resorption (family history, previous trauma, thin roots). However, aligners have limitations in complex malocclusions requiring extensive tooth movement, where fixed appliances may still be necessary [15]. CBCT-based evaluations have consistently demonstrated that patients treated with clear aligners experience a lower prevalence and severity of apical root resorption compared with those receiving fixed appliances, highlighting the biomechanical advantages of intermittent, lighter forces in preserving root integrity [16].

## Conclusion:

Fixed orthodontic appliances induce significantly greater root resorption compared to clear aligners. Maxillary incisors were most affected, followed by premolars and molars. Thus, CBCT imaging enabled precise volumetric evaluation, highlighting aligners as a safer alternative for minimizing apical root loss. Hence, treatment planning should consider individual susceptibility to optimize patient outcomes.
